# Athlete’s Hepatitis: A Case of a Young Healthy Male Developing Hypoxic Hepatitis After a Half-Marathon

**DOI:** 10.7759/cureus.77491

**Published:** 2025-01-15

**Authors:** Joel Gabin Konlack Mekontso, Akil Olliverrie, Joshua Diaz, Sara Samad, John Trillo

**Affiliations:** 1 Internal Medicine, NYC Health + Hospitals, South Brooklyn Health, Brooklyn, USA; 2 Gastroenterology, NYC Health + Hospitals, South Brooklyn Health, Brooklyn, USA

**Keywords:** acute liver injury, cardiogenic ischemic hepatitis, exertional heat stroke, extreme exercise, rare diagnosis

## Abstract

Athlete’s hepatitis is a rare form of ischemic hepatitis caused by hypoxic liver injury during extreme physical exertion. We present the case of a 25-year-old healthy male who developed severe transaminitis after completing a half-marathon. He presented with syncope, hypotension, and hyperthermia, followed by markedly elevated liver enzymes. An extensive evaluation ruled out viral, toxic, and structural causes of liver injury. Conservative management led to full recovery, with liver enzymes normalizing within one week. This case highlights the liver's susceptibility to ischemic injury during intense exercise due to blood flow redistribution to active muscles. Although rare, athlete’s hepatitis should be considered in the differential diagnosis of acute liver injury in athletes. Early recognition and supportive care are essential for favorable outcomes.

## Introduction

Ischemic hepatitis results from impaired perfusion of the hepatic parenchyma, leading to hypoxic hepatocyte injury. It is commonly seen in critically ill patients with conditions such as decompensated heart failure, severe respiratory failure, or septic shock, where oxygen delivery to the liver is significantly compromised [1]. Athlete’s hepatitis, a rare subset of ischemic hepatitis, occurs in otherwise healthy individuals and is triggered by prolonged, intense physical exertion. It reflects the liver’s unique vulnerability to hypoxic injury during extreme exercise. Here, we present the case of a young, healthy male who developed ischemic hepatitis following the completion of a half marathon. This article is reported according to Case Report (CARE) guidelines [2].

This work was presented as a poster at the American College of Gastroenterology's conference on October 23, 2023, in Vancouver, Canada.

## Case presentation

This case involves a 25-year-old Caucasian male with no notable medical or family history who presented to our Emergency Department on June 19, 2023, after experiencing syncope upon completing 11 miles of his first-ever half-marathon. He was under temperatures of 75℉ (23.9°C) and 60% humid weather. On arrival, he was found to be hypotensive with systolic blood pressure in the 70s, hyperthermic with the highest recorded temperature of 104.1°F (40°C), tachycardic (112 beats per minute), and had a BMI of 24. His capillary blood glucose level was 80 mg/dL. An electrocardiogram showed sinus tachycardia without ischemic changes. Initial management included cooling measures (ice packs and cooling blankets), continuous temperature monitoring, and intravenous hydration with cold fluids (2 litres of Ringer lactate bolus followed by 100 mL/min for 10-12 hours).

Renal and hepatic chemistries were within normal limits, and other pertinent laboratory findings are summarized in Table 1. Based on these findings, the patient was admitted with a working diagnosis of syncope secondary to exertional heat stroke. Drug use or overdose was also considered as a differential diagnosis; however, the patient denied alcohol, tobacco, and substance use, and the urine drug screen was negative.

**Table 1:** Pertinent laboratory findings on admission.

Laboratory tests	Results	Normal range (< / =)
D-dimer (ng/mL)	898	350 (ng/mL)
Creatine phosphokinase (U/L)	298	190 (U/L)
High-sensitivity troponin (ng/L)	80	22 (ng/L)

By hospital day two, liver enzymes showed a marked increase: Alanine transaminase (ALT) rose from 17 U/L to 3663 U/L, and aspartate transaminase (AST) from 33 U/L to 2516 U/L, with no other clinical finding prompting for a gastroenterology consultation. Given the clinical context, a preliminary diagnosis of athlete’s hepatitis was made, although alternative etiologies, including acute viral and toxic hepatitis, were thoroughly investigated. A comprehensive serologic workup for viral hepatitis and other common causes was negative. No electrolyte disturbances were observed, and a liver ultrasound revealed a homogeneous hypoechogenic hepatic parenchyma with no evidence of structural abnormality. The patient was managed conservatively with continued hydration and serial monitoring of liver enzymes. He received N-acetyl cysteine 150 mg/kg over one hour (total 12.0 g), then 12.5 mg/kg over four hours (total 1.0 g), followed by 6.25 mg/kg over 67 hours (total 500 mg). His transaminases steadily improved, returning to baseline within one week of admission. The patient was discharged, and the follow-up was uneventful.

## Discussion

Heatstroke is a life-threatening condition characterized by the body's core temperature exceeding 40°C (104°F). It often results from intense physical activity in hot environments but can also affect vulnerable individuals even at rest. It occurs as a multifactorial process resulting from an imbalance in heat generation (strenuous exercise) and dissipation (heavy clothing, high ambient temperatures, and high humidity). Healthy adolescents and adults with occupations that involve heavy exertion (athletes, military personnel, and firefighters) are at elevated risk. Viral or bacterial infections, as well as recreational drug use, also increase the risk [3-5]. Heatstroke’s pathophysiological effects, including systemic hypoperfusion and rhabdomyolysis, create conditions that can precipitate ischemic or hypoxic hepatitis by compromising hepatic oxygenation and causing direct hepatocyte injury.

Ischemic or hypoxic hepatitis accounts for over 50% of cases of massive liver enzyme elevation (>10 times the upper limit of normal) in hospitalized patients, followed by pancreaticobiliary disease, drugs and toxins, and viral hepatitis [6,7]. In the setting of exertional heatstroke, hypoxic hepatitis is triggered by factors such as hypoperfusion, rhabdomyolysis, and thermal damage, with high outdoor temperature and humidity exacerbating these effects. Significant elevations in biomarkers such as creatine kinase, transaminases, blood urea nitrogen, serum creatinine, and lactate dehydrogenase reflect the extent of injury [8]. Monitoring of cardiac troponins and D-dimer can guide early recognition of myocardial injury and coagulation abnormalities. While physical exercise is generally beneficial for liver health, risks arise with inadequate hydration, extreme exertion, or behaviors like anabolic steroid use or unprotected travel to hepatitis-endemic areas [9]. This highlights the dual nature of exercise-health-promoting under controlled conditions but potentially harmful in extreme circumstances.

The management of athlete's hepatitis involves addressing both the underlying heatstroke and the liver injury it causes. The primary intervention should focus on active cooling, even before transport is initiated [10]. Advanced life support should be promptly initiated when warranted. The cooling goal is a core body temperature<39°C (102.2°F) within 30 minutes and is best achieved through conductive cooling (ice water bath immersion). Despite this preference, evaporative and convective cooling must be promptly started should conductive measures be unavailable, as in our scenario, although the cooling rate is slower [3,4]. Continuous temperature monitoring is recommended alongside intravenous fluid replacement and electrolyte rebalancing. Antipyretics (acetaminophen and NSAIDs) interfere with the hypothalamic set point change caused by pyrogens and are ineffective in heatstroke. They may even be harmful, as they carry a risk of further liver, kidney, and gastrointestinal injury, as well as bleeding [4,5]. Benzodiazepines may also be considered for patients experiencing shivering or seizures [11-13]. Active cooling should be continued until core body temperature normalizes and neurological stability is achieved.

For the liver injury itself, supportive care is paramount. Hydration remains crucial to ensure adequate perfusion and prevent further hepatic injury. In our case, treatment with N-acetylcysteine (NAC) was initiated despite the absence of evidence for acetaminophen toxicity. NAC’s antioxidant properties, replenishing glutathione, and neutralizing reactive oxygen species make it a plausible therapeutic option for ischemic hepatitis [14]. By mitigating oxidative stress, NAC can potentially limit hepatocyte damage and promote recovery. Although its routine use in ischemic hepatitis is not established, it was considered reasonable given the marked transaminase elevations and the potential oxidative stress in our patient. 

The prognosis for athlete’s hepatitis is generally favorable with timely recognition and appropriate care, although rare cases of acute liver failure may require liver transplantation [15]. Prevention involves staying hydrated, acclimatizing gradually, wearing appropriate clothing, scheduling activities for cooler times, taking breaks in the shade, and monitoring for symptoms [3-5]. By understanding the risk factors, recognizing the symptoms, and taking preventive measures, we can reduce the incidence of athlete’s hepatitis and protect ourselves and others from this potentially grave pathology.

## Conclusions

Athlete’s hepatitis is a rare but important diagnosis to consider in athletes presenting with acute liver enzyme abnormalities post-exertion. This condition, often associated with strenuous activities such as marathons, can mimic other causes of liver dysfunction, leading to potential misdiagnosis. Increased awareness among clinicians and sports medicine practitioners can facilitate timely diagnosis, promote effective management strategies, and reduce the risk of complications, ultimately ensuring well-being.
